# An autonomous snapper featuring adaptive actuation and embodied intelligence

**DOI:** 10.1126/sciadv.adu4268

**Published:** 2025-04-04

**Authors:** Duygu S. Polat, Zihua Chen, Samüel A. M. Weima, Satoshi Aya, Danqing Liu

**Affiliations:** ^1^ Human Interactive Materials, Department of Chemical Engineering and Chemistry, Eindhoven University of Technology, Den Dolech 2, 5612 AZ Eindhoven, Netherlands.; ^2^ Institute for Complex Molecular Systems, Eindhoven University of Technology, Den Dolech 2, 5612 AZ Eindhoven, Netherlands.; ^3^ South China Advanced Institute for Soft Matter Science and Technology (AISMST), School of Emergent Soft Matter, South China University of Technology, Guangzhou, China.; ^4^ Guangdong Provincial Key Laboratory of Functional and Intelligent Hybrid Materials and Devices, Guangdong Basic Research Center of Excellence for Energy and Information Polymer Materials, South China University of Technology, Guangzhou, China.

## Abstract

Developing artificial systems with autonomous motion is essential for creating devices that emulate nature’s adaptive mechanisms. Here, we introduce a light-driven liquid crystalline network snapper that integrates both sensing and actuation capabilities, enabling adaptive responses to environmental conditions. Under constant light illumination, the snapper undergoes spontaneous snap-through transformation driven by the elastic instability embedded within the material. The snapper achieves out-of-equilibrium motion through continuous energy transfer with the environment, enabling it to sustain dynamic, reversible cycles of snapping without external control. We demonstrate the ability of the liquid crystalline network snapper to detect environmental changes—such as shifts in temperature, surface roughness, and color—demonstrating a form of embodied intelligence. This work offers a distinctive strategy for designing biomimetic devices that merge embodied intelligence with autonomous motion, opening pathways for advanced, adaptive systems for soft robotics.

## Teaser

Embedded intelligence enables liquid crystal snappers to achieve out-of-equilibrium motion under continuous light illumination.

## INTRODUCTION

The concept of autonomy is one of the key strategies that animals and plants take to maintain and regulate their motions independently while adapting to external changes to survive and function (*1*). The natural world offers valuable insights for designing active materials that replicate the dynamic and adaptive characteristics of living organisms (*2*, *3*). Achieving true autonomy in engineered systems necessitates materials that are not only responsive but also capable of self-regulating behaviors, allowing for dynamic interactions with their surroundings (*4*, *5*). A primary challenge in this area is the development of active materials that can operate out of equilibrium with a stable energy supply while simultaneously adapting to and engaging with their environment (*6*). Unlike natural systems, which have evolved to seamlessly integrate sensing, processing, and actuation, the integration of these functions within engineered systems remains a major hurdle (*7*).

Among various actuation mechanisms for active materials, instability-driven systems offer a promising approach for enhancing response speed and adaptability (*2*, *8*–*11*). Elastic structures exhibiting bistability, characterized by two distinct equilibrium states, are prevalent in both natural and engineered systems, such as the quick snap of the carnivorous plant Venus flytrap and the precision of hummingbird beaks. Snap-through instability facilitates rapid transitions between two stable states, allowing hummingbirds to capture insects in midair (*12*) and Venus flytraps to surpass the constraints of their intrinsically slow and limited movement to catch prey (*13*). Recently, by harnessing elastic instabilities in active materials, leaping actuators (*14*–*19*), highly efficient swimmers (*20*–*22*), and gait-like locomotors (*23*–*25*) have been developed. While the natural systems are self-regenerative (*26*), their engineered counterparts are limited to a single snap-through event without active control (*27*–*30*) due to the inherent energy barrier between the stable states (*31*). This poses a challenge for achieving self-sustained autonomous motion, as it requires feedback-driven interactions between structural deformation and energy dissipation under a constant energy supply (*32*).

To introduce self-regulating motion, engineered systems often rely on temperature gradients from heated surfaces (*24*, *33*, *34*). The work of Zhao *et al.* (*33*) is particularly relevant to this work, introducing self-sustained snapping under constant illumination driven by a heated surface. As the surface both drives and shapes the motion, the active material’s behavior is largely reactive rather than adaptive, as it continually responds to the surface’s changes rather than independently adjusting its behavior based on broader conditions or stimuli. Another approach for self-sustained motion relevant to this work was introduced by Gelebart *et al.* (*35*), demonstrating locomotion under constant illumination using the interaction with the surface. In this work, the surface merely acts as a boundary condition because the motion is driven by a self-shadowing mechanism. For true adaptability, an active material needs to benefit from an independent control mechanism—such as an external stimulus that drives its out-of-equilibrium motion, allowing dynamic interactions with the environment.

The autonomous snapper consists of a liquid crystal network (LCN)—a class of smart materials that exhibit reversible shape changes in response to various external stimuli (*35*–*44*)—combined with a thermal modulation layer (TML) to achieve reversibility through energy dissipation. We fabricate the LCN with an active, splay-bend aligned segment in the center and a passive, isotropic segment near the edges. Upon heating, the partially active LCN develops a preferred curvature in the central section, causing the arch to snap to the opposite position. Because the actuation pattern is programmed directly into the LCN during fabrication, this snapping behavior is highly robust and reliable. Contact with the TML causes the LCN to release strain energy, prompting it to snap back to its original state and generating negative feedback. While the actuation of the LCN drives the snapping, the TML functions as an environmental boundary condition rather than an active external control to achieve reversibly. This design mirrors the self-regulating motions found in nature, where organisms depend on external cues to sustain their cycles and establish autonomy through an interdependent relationship with the environment (*45*). The snapper’s adaptive, autonomous motion exemplifies “embodied intelligence,” where system behavior emerges from physical interactions with the environment without the need for centralized control (*46*, *47*). Our findings indicate that the snapper can differentiate environmental factors such as color, texture, and temperature, effectively functioning as both an actuator and a sensor. In addition, the instability-driven mechanism enables rapid actuation, overcoming the usual limitations in response speed found in conventional soft materials (*9*). The autonomous snapper, thus, represents an innovative approach to achieving responsive, adaptive systems for soft robotics capable of sensing and adjusting to environmental changes in real time.

## RESULTS

### Fabrication of LCN with encoded elastic instability

**Fig. 1.**
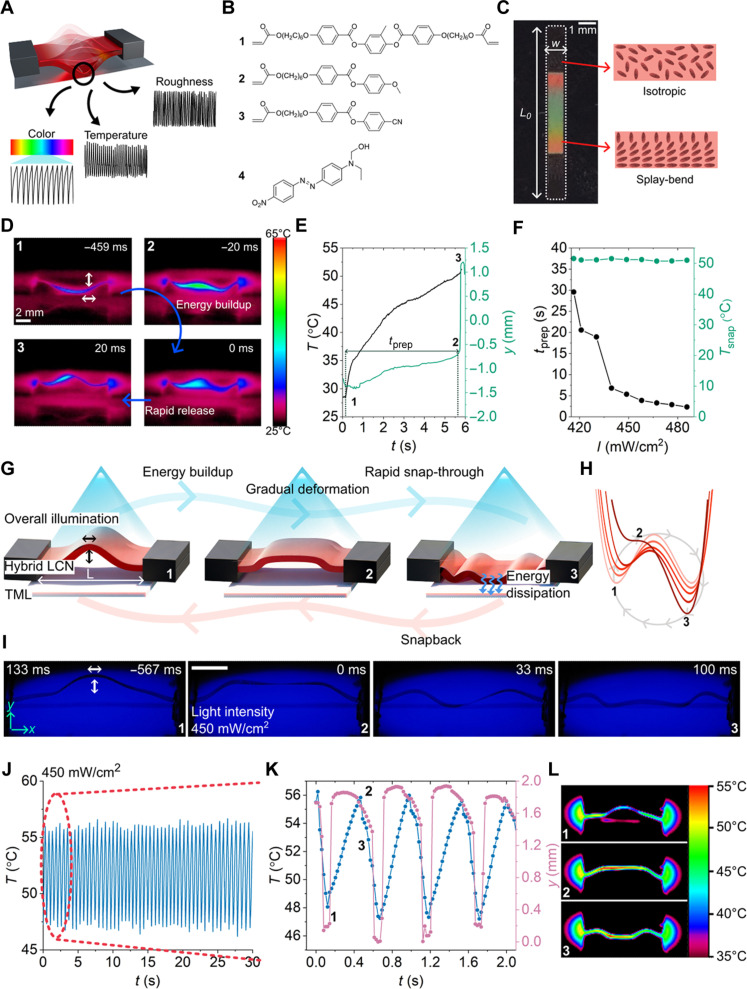
Concept and design of the autonomous snapper. (**A**) Illustration of the autonomous snapper. Self-sustained snapping is achieved through the interaction of the partially active LCN with TML. Signal characteristics of the self-snapping depend on the surface properties of the TML (color, temperature, and roughness). (**B**) Chemical components used for the fabrication of the LCN. **1**, diacrylate monomer; **2** and **3**, monoacrylate monomers; **4**, photothermal dye. (**C**) Photograph of the LCN between cross-polarizers showing the active (birefringent) and passive segments (nonbirefringent). (**D**) Snapshots of LCN at different states of snap-through. (**E**) Snap-through kinetics of the LCN upon illumination with 455-nm light. (**F**) Influence of light intensity on the preparation time and temperature of snap-through. (**G**) Schematic illustration of the self-sustained snapping. (**H**) Transformation of the energy landscape during self-sustained snapping. (**I**) Snapshots of autonomous snapper at different states of self-sustained snapping. State 1, immediately after illumination, LCN gains slight curvature; state 2, LCN has gained critical strain energy to overcome the energy barrier; state 3, LCN after snap-through. Scale bar, 2 mm. (**J**) Temperature oscillations at the center of the snapper during self-snapping. (**K**) Time evolution of displacement and temperature during self-snapping and (**L**) corresponding infrared (IR) images of the autonomous snapper.

Autonomy in natural systems depends on out-of-equilibrium dynamics, which involve continuous energy dissipation and are regulated through feedback loops (*4*). Inspired by nature’s approach to autonomy, we designed a snapper capable of continuously storing and dissipating energy while self-regulating its snapping motion through embodied intelligence (Fig. 1A). The main component of the autonomous snapper is a partially active LCN. We prepared the LCN by two-step masked photopolymerization of LC monomers **1**, **2**, and **3** in the presence of photothermal dye **4** with an absorption peak at 455 nm (Fig. 1B and fig. S1). In the first step, we photopolymerized only the center of the LCN through a photomask at the nematic (N) state to freeze the splay-bend alignment, where the surface anchoring changes from homeotropic to planar. Once the central part was polymerized, we heated the LC monomer mixture above the isotropic-nematic (Iso-N) transition temperature and photopolymerized the sample at two ends. These operations lead to an LCN with controlled splay-bend N and Iso regions (Fig. 1C). We fix the ratio of the splay-bend N region as 0.5 to ensure strong snap-through instability (*48*).

To demonstrate the encoded instability of the partially active LCN (*w* = 0.75 mm, *L*_0_ = 10 mm, and *t* = 20 μm), we fix the length between the two ends to be *L* = 9.8 mm to create a convex-shaped ribbon with top surface exhibiting planar anchoring (Fig. 1D). Geometry of the snapper was selected so that the bending and stretching deformations are strongly coupled and bistability is favored (*9*, *13*, *49*). By introducing both active and passive segments into the LCN during fabrication, we break the top-bottom symmetry. When the LCN is buckled and stimulated, this design creates a bias, causing the strip to favor the top configuration. In contrast to previous approaches, where a bias is introduced by applying a thickness gradient of stimulation (*14*, *15*, *24*, *27*, *33*), this method encodes the bias directly into the design. This allows the strip to exhibit instability under uniform stimulation, without the need for a spatially varying stimulus (*48*). Upon overall illumination with 455-nm light, photothermal dye **4** absorbs the light and dissipates the heat to the polymer network. Photothermal heating disrupts the molecular order, which leads to expansion and contraction on the homeotropic and planar sides, respectively. As a result, the preferred curvature of the active segment becomes negative. The passive segments serve two main purposes: They eliminate the stress at the edges and, second, they do not attain preferred curvature, remain immobile, and, instead, cause the energy barrier to increase with increasing stimulation. When the stimulation is sufficient to achieve the threshold strain for snap-through [preferred curvature (κ) = 0.53 mm^−1^; fig. S2], the energy barrier disappears, making the bottom configuration unstable and the strip exhibits a rapid snap-through toward the light source (Fig. 1E) (*48*). We define the time for the onset of instability following the start of illumination as the preparation period, *t*_prep_, which exhibits strong dependency on the light intensity (Fig. 1F). However, the temperature of the LCN at the moment of snap-through is not influenced by the light intensity, as it is predefined by the geometry (LL0) and threshold strain for snap-through (figs. S3 and S4). Snap-through toward a light source further reinforces the energy landscape and stabilizes the snapped state (Fig. 1D). Introducing autonomy, therefore, requires a strategy that can reset the energy landscape.

### Establishing self-sustained snapping

Our approach to achieving autonomous motion involves a design that enables the continuous modulation of the energy landscape. In addition to the snapper, as shown in Fig. 1G, the design includes a TML, a white paper substrate, that can absorb the dissipated energy from the snapper. We initially create a concave-shaped ribbon with planar alignment at the top surface. We then induce a positive preferred curvature to cause a snap-through away from the light source and toward the TML when the light intensity is sufficient to overcome the energy barrier (fig. S5). Upon contact with the TML, the LCN loses strain energy, which makes the convex shape unstable. This interaction causes the energy landscape to switch to the initial state as depicted in Fig. 1H and provides negative feedback to the LCN. Upon snap back toward the light source, the LCN starts to attain a concave shape, resetting the motion. Switching of the energy landscape through the interaction of the bistable LCN with the TML establishes a self-sustained motion (Fig. 1, I and J, and movie S1). To better understand the dynamics of autonomous snapping, we examine the time evolution of the maximum *y* coordinate and surface temperature of the snapper (Fig. 1, K and L). The initial phase of self-sustained snapping (state 1 to state 2) corresponds to the preparation period for snap-through, followed by a rapid snap-through (state 2 to state 3). Meanwhile, the subsequent phase (state 3 to state 1) represents the snap-back kinetics, driven by the energy transfer rate between the snapper and the TML (fig. S6). The snapper can sustain its self-oscillation for long periods (~50 min) with no sign of a decrease in its frequency (fig. S7).

### Dynamics of autonomous snapping

**Fig. 2.**
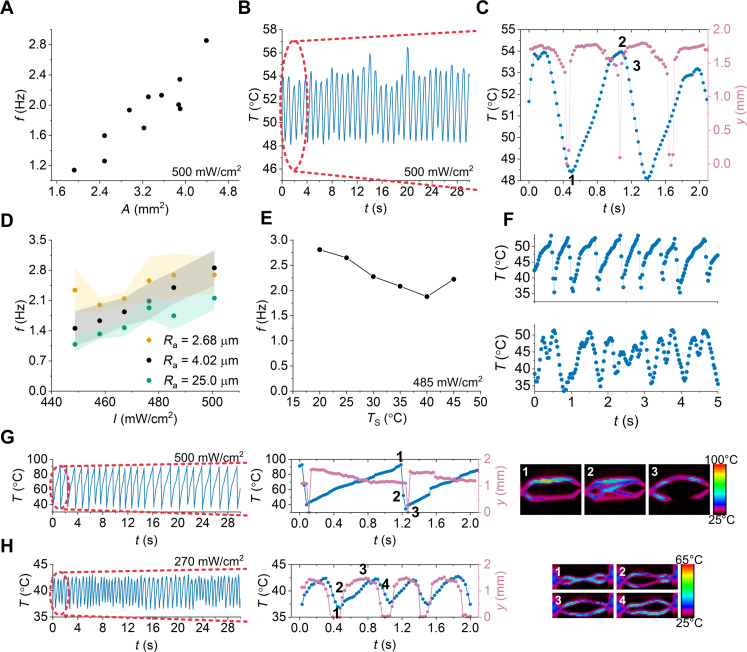
Exploring the embodied intelligence of autonomous snapper and material requirements for self-snapping. (**A**) Influence of the heat transfer area of the snapper with the thermal energy modulation layer on the frequency of oscillations. (**B**) Temperature oscillations of the snapper with 0.880-mm elevation from the TML with a corresponding *A*_C_ of 2.2 mm^2^. (**C**) Temperature and displacement evolution of self-sustained snapping. (**D**) Influence of *R*_a_ of TML on the frequency of self-snapping. (**E**) *T*_S_ dependency on the frequency of self-sustained snapping. (**F**) Temperature oscillations of the snapper for *T*_S_ of 23.5°C (top) and 45°C (bottom). Time evolution of displacement and temperature of (**G**) D-LCN and (**H**) L-LCN during self-snapping with corresponding IR images.

Understanding the dynamics of self-sustained snapping is crucial for optimizing the performance of the snapper in various applications. The snap-back kinetics, or the rate at which the LCN returns to its original state, are primarily governed by the dissipation rate to the TML. To quantitatively describe this relationship, we consider the energy accumulation of the LCN from state 3 to state 1 (Fig. 1G)

$$C_{p}m\frac{\partial T}{\partial t}=\dot{Q}-h2A_{s}\left(T-T_{0}\right)-\frac{kA_{c}\left(T-T_{s}\right)}{L}$$

where $C_{p}$, m, $A_{s}$, and k are specific heat capacity, mass, surface area, and thermal conductivity, respectively, of the LCN. $\dot{Q}$, h, $A_{c}$, $T_{0}$, and $T_{s}$ denote photothermal heat absorbed by the LCN, convective heat transfer coefficient, heat transfer area, ambient temperature, and surface temperature, respectively. The equation can be rewritten as

$$\frac{\partial T}{\partial t}=M+\mathrm{NT}$$

where $N=-\frac{h2A_{s}+\frac{kA_{c}}{L}\;}{C_{p}m}$ and $M=-\frac{\dot{Q}+h2A_{s}T_{0}-\frac{kA_{c}}{L}T_{s}\;}{C_{p}m}$. Solving the derivative gives

$$T\left(t\right)=\frac{\left(NT_{0}+M\right)}{N}e^{\mathrm{Nt}}-\frac{M}{N}$$

showing an exponential decay of temperature that we can control by altering $\dot{Q}$, $A_{c}$, and $T_{s}$. To experimentally verify the importance of the parameters, we first test the influence of $A_{c}$ on the frequency of self-sustained snapping. As expected, higher $A_{c}$ leads to faster oscillations because it increases the decay constant (Fig. 2A, fig. S8, and movie S6). For smaller $A_{c}$, the rate of cooling down becomes comparable with *t*_prep_, leading to a more symmetrical response in both the temperature and displacement curves (Fig. 2B). Figure 2 shows an example of the corresponding time evolution of the snapper temperature self-oscillating at 1 Hz at a heat transfer area of 2 mm^2^. The experimental setup also has an influence on this change because we controlled the heat transfer area by adjusting the vertical distance between the LCN and the TML. Although the velocity of the LCN remains relatively unchanged, this adjustment contributes to the decrease in frequency, as the LCN needs to achieve a larger displacement between state 3 and state 1.

To further understand the dynamics of self-sustained snapping, we explored the impact of the design of the TML surface. Our observations suggest that increasing the roughness average ($R_{a}$) of the TML surface decreases the self-snapping frequency (Fig. 2D and fig. S9). This may arise because a higher $R_{a}$ leads to a reduction of $A_{c}$, thereby slowing the cooling rate. Next, we investigated the influence of the temperature gradient ($T-T_{s}$). For $T_{s}$ values below 40°C, the self-snapping frequency increases with decreasing temperature, presumably due to the increase in the cooling rate (Fig. 2E and movie S2). However, when $T_{s}$ is 45°C, we observed a higher frequency, which could be explained by the rubbery state of the LCN (fig. S10). At the rubbery state, the motion of the LCN is highly influenced by gravity, which decreases the threshold strain for snap-through and *t*_prep_ (Fig. 2F). When the $T_{s}$ is higher than 45°C, the LCN can exhibit snap-through; however, upon contacting the TML due to inefficient cooling rate, it cannot snap back to the initial state.

While the control of the thermal dissipation is crucial for the autonomous snapping as discussed above, the elastic properties of LCNs also play a dominant role in dictating deformational pathways. To control the elasticity of LCNs, we fabricated LCNs with various cross-linking densities by adjusting the concentration of monomer **1**. The concentration of monomer **1** was selected to span from the minimum weight percentage required for network formation at one end to a network composed entirely of monomer **1** at the other end. From our experimental observation, the cross-linking density alters both the energy barrier and the timescale for snap-through (Fig. 2, G and H, and movie S3). Change in the energy barrier is evident when comparing the intensity of the light required to initiate snap-through. While the LCN with 97.5 wt % of monomer **1** (D-LCN) does not exhibit self-sustained snapping at light intensities below 460 mW/cm^2^, snap-through of LCN with 9.75 wt % of monomer **1** (L-LCN) can be photoinitiated at 245 mW/cm^2^ (fig. S11). The increase in the energy barrier can be explained by the fact that the storage modulus of L-LCN at the moment of snap-through (125 mPa) is half of the storage modulus of D-LCN (280 mPa) (fig. S10). As a result, the motion of L-LCN is highly influenced by gravity, which reduces the threshold strain for snap-through. This influence is also evident when comparing the preferred curvatures of the LCNs at the snap-through temperature, which is the highest for D-LCN (fig. S2). In addition, a higher cross-linking density enhances the time for snap-through, which scales in proportion to $∼\;\frac{1}{B}$, where B is the bending stiffness (fig. S11) (*9*, *50*).

### Understanding the mechanism of snapping via modeling and simulation

**Fig. 3.**
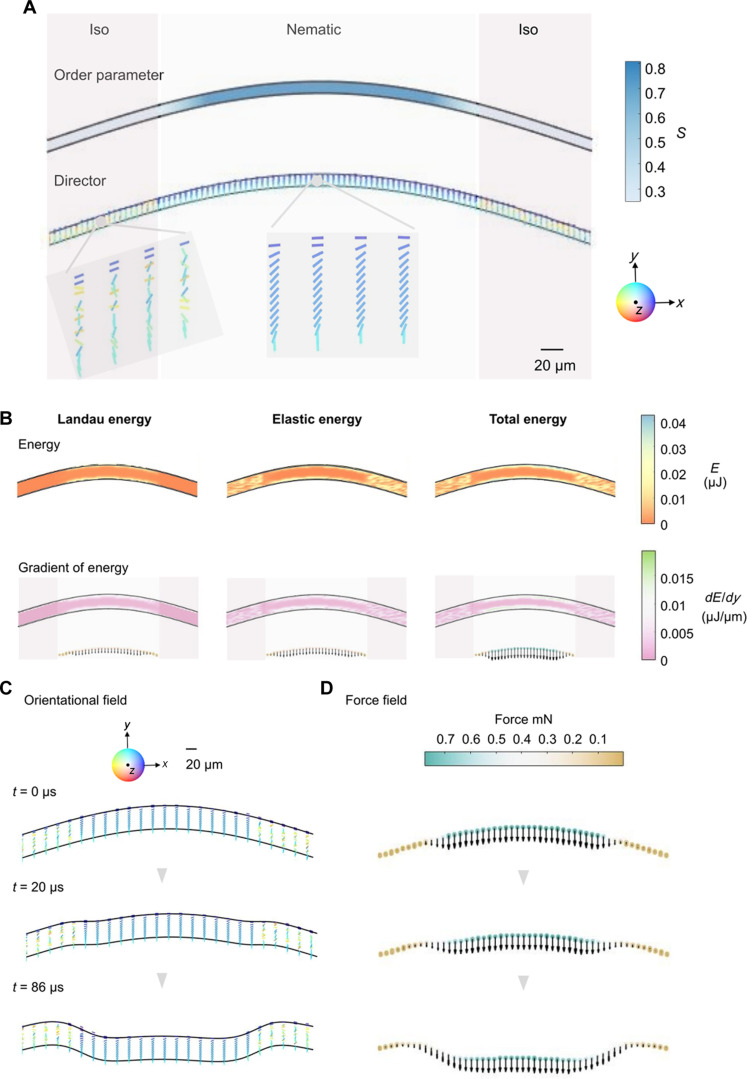
Simulation and analysis of LC director, order parameter, energy distribution, and shape deformation process of the snapper. (**A**) The snapper is divided into three main regions: the Iso phases and the N phase. Under the influence of elastic energy, Landau energy, and surface anchoring energy, we observe the equilibrium distribution of the director within the snapper. To better depict the transition between the Iso phase and the N phase, we use the order parameter as a physical quantity. Within a range of approximately 20 μm, the order parameter transitions continuously from 0.3 to 0.8, delineating the boundary between the two phases. (**B**) The distribution of Landau energy and elastic energy within the snapper, as well as the overall energy distribution, which is the sum of both (top). The distribution of energy gradients indicate the rate of change of the energy within the snapper (bottom). Changes in energy gradients reflect variations in the magnitude and direction of forces acting on LC molecules at different positions. In simulating the shape transformation of the snapper under the influence of elastic energy and membrane surface tension, we focus on the orientational field of LC molecules and the dynamic variation of the force field. (**C**) The elastic constant of the LC is set to 5 pN. (**D**) In addition to elastic energy, membrane surface tension notably affects the shape transformation of the snapper. In this simulation, the surface stretching coefficient (*K*_str_) is set to 1 × 10^−5^ J·m^−2^, and the bending coefficient (*K*_curv_) is set to 1 × 10^−5^ J·m^−2^. In the force field, forces in the N phase region are substantially greater than those in Iso phase regions, directed downward. This difference results in a greater downward thrust on the snapper in the N phase, prompting it to move downward. The deformation process lasts for 86 μs, during which gradual shape changes occur.

To establish relationships between material parameters and snapping behaviors and to see the physical processes closer, we have built a simulation system based on a *Q*-tensor–coupled bead-spring network model that takes account of the elasticities of the polymer network and the LC entities (*51*–*53*). While the positional-dependent LC order parameter and orientations in the LCN are encoded in each bead site, the elastic properties, including the aforementioned bending stiffness, are embodied as the elasticities of the spring network. The numerical calculation provides a facile way to compute and estimate properties of either the pure LCN or the partially active LCN, giving a semiquantification of physical parameters (*54*–*56*). To construct a partially active LCN system that comprises splay-bend alignment in the center and isotropic at the edges as designed in the experiments, we use different Landau coefficients for the center and edges. The equilibrium order parameter, $S_{\text{bulk}}=\frac{1}{4}\left(1+3\sqrt{1-\frac{8}{3U}}\right)$, is 0.81 and 0.25 for the splay-bend N and Iso regimes, respectively (*51*, *57*). We approximate the LCN as a two-dimensional (2D) concave-shaped ribbon because no deformation occurs along the *y* axis and created the initial geometrical conditions of its shape similar to the experimental setting (Fig. 3A). By setting strong homeotropic and planar anchoring on the top and bottom surfaces, respectively, we first relax the system to equilibrium. The middle part with nematicity realizes the splay-bend deformation, and the Iso state is obtained at two edge parts (Fig. 3A). In the following, we set the transitional length between the Iso and the N as 50 μm at the Iso-N boundary. In experiments, the length of the transitional area, say d, depends on the spatial resolution of the photopolymerization, usually in a micrometer scale. When the spacing d varies, corresponding to a sudden vanishing of the LCN order at the Iso-N boundary, the force arising from the order parameter gradient changes and thereby slightly modifies the dynamics (fig. S12). As seen in Fig. 3 (A and B) and fig. S13, the spatial variations of orientation and order parameter cost free energy and serve as sources of generating force. In bulk nematic of the middle part, the gradient of orientation in the splay-bend director field leads to forces (*52*, *53*). No orientational force arises in the Iso regime. Meanwhile, a fast drop of the order parameter at the Iso-N interface creates a boundary force. Because of the continuous transition of order parameter over several micrometers, this boundary force is not large and continuously vanishes when going toward the Iso side. As a result, the force distribution drags the LCN downward and triggers its snapping motion, as observed in the experiments. Figure 3A demonstrates the time evolution of both the orientational and force fields upon the snapping motion. The orientational field is nearly unchanged with time within the LCN, which is realized by strong anchoring in the simulation. This leads to the force field also not unchanging. Once the LCN touches the TML, the motion will stop as found in the experiments.

### Embedded intelligence of autonomous snappers

**Fig. 4.**
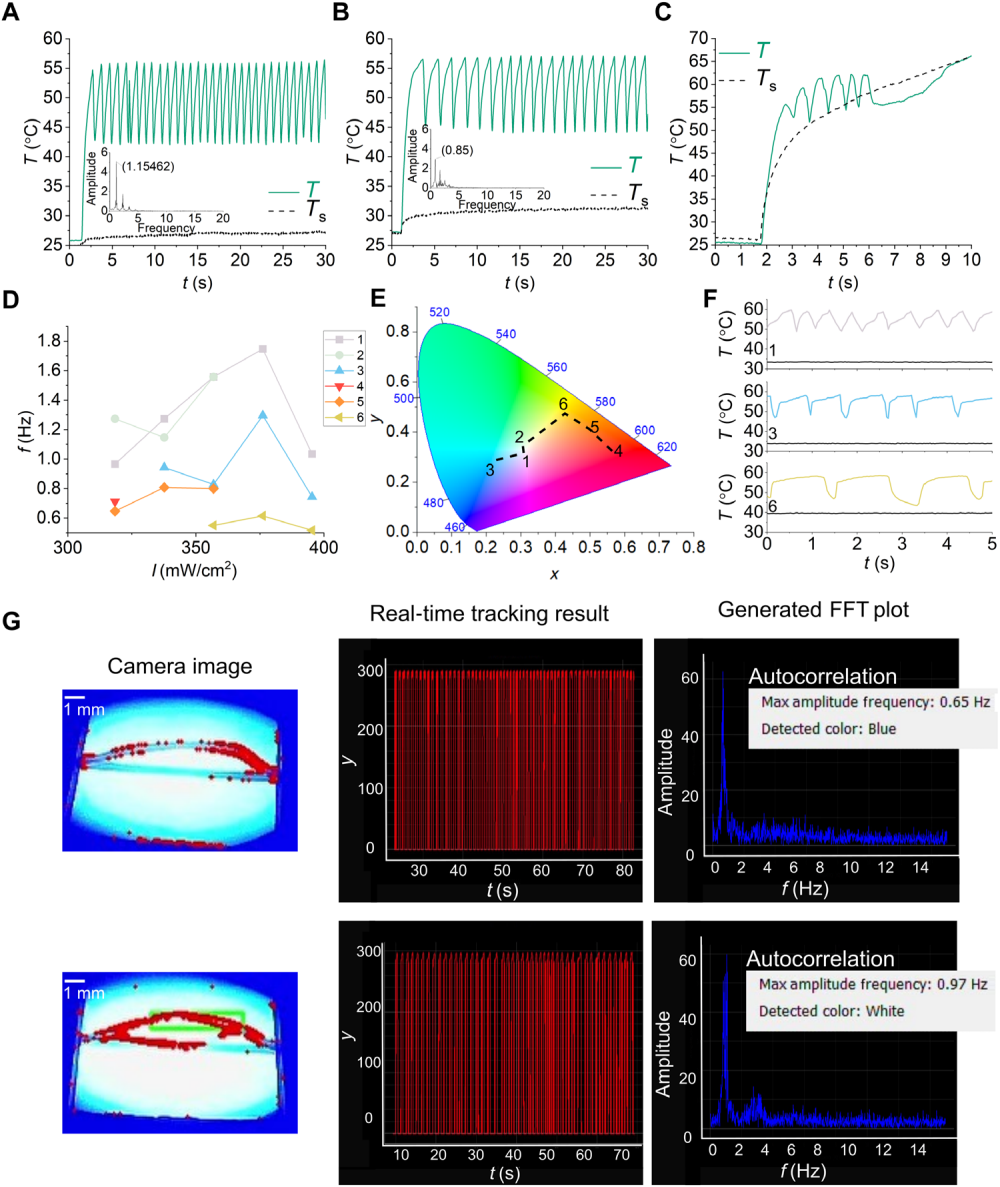
Demonstrating the embodied intelligence of autonomous snapper. Temperature oscillations on a white (**A**), blue (**B**), and black (**C**) TML surface during self-sustained snapping. (**D**) Influence of TML color on the frequency of self-snapping. (**E**) Corresponding locations of the TML surfaces on the color map. (**F**) Temperature oscillations of the snapper on TML surfaces 1, 3, and 6. Black lines correspond to $T_{s}$. (**G**) Snapshots taken while the Python script was autocorrelating the color of the blue (top) and white (bottom) TML surface through the frequency of self-sustained snapping. FFT, fast Fourier transform.

The autonomy of the snappers can be immediately diversified to various self-feedback applications by design. Here, we showcase a color detection application using the variations in the self-sustained snapping behavior of the LCN on the TML surfaces of different colors (Fig. 4, A to C). When the TML surface color is changed from white to blue, the frequency of self-sustained snapping decreases by 26%. This reduction is attributed to the higher absorption of 455-nm light by the blue TML surface, leading to an increase in $T_{s}$ (Fig. 4B). In addition, on a black TML surface, the LCN snaps only a few times before $T_{s}$ reaches 58°C (Fig. 4C). As a result, the convex shape of the LCN becomes stable, and the self-sustained snapping motion stops. Consequently, the snapper can exhibit different behaviors depending on the color of the TML (Fig. 4, D to F).

After developing this functionality, leveraging the embodied intelligence of the snapper, we can use it to function both as a sensor and as an actuator. We developed a Python script that detects the movement of our snapper and draws a bounding box around the detected moving object. The time evolution of the maximum *y* coordinate of the bounding box is subjected to a Fourier transform, of which the peak with the highest intensity is characteristic for a combination of light intensity, snapper type, and TML color. We recorded the peak frequency for a consistent batch of snappers and fixed light intensity (440 mW/cm^2^) and used this dataset to define the frequency ranges that correspond to white and blue substrates, respectively. Subsequently, we successfully used the script to detect substrate color, as shown in Fig. 4G and movie S5.

## DISCUSSION

In this work, we introduce a design framework for achieving autonomy in bistable systems through embodied intelligence. Our approach is realized in snappers that exhibit self-sustained oscillations, driven by photoinitiated snap-through behavior and controlled energy dissipation to the environment. Constructed from partially active LCNs, these snappers can be fine-tuned to produce specific self-oscillation patterns by adjusting their cross-linking density. Much like natural systems, these snappers operate out of equilibrium under static stimuli, enabling them to respond dynamically and adaptively to environmental changes. Here, light serves as the primary trigger for autonomous motion, while the frequency and characteristics of self-snapping are determined by the interdependent relationship between the snapper and its surrounding environment, a relationship mirrored by the TML. Functioning as both actuator and sensor, the snappers can operate within a frequency range of 0.5 to 3.5 Hz at mild temperatures below 55°C. The compact design of these autonomous snappers, with integrated sensing and actuation capabilities, makes them particularly suitable for soft robotics applications, where they could enhance the interaction between robotic systems and their environment. We believe that this approach, leveraging embedded instability and embodied intelligence, opens an avenue for designing biomimetic devices with autonomous capabilities.

## MATERIALS AND METHODS

### Materials

The reactive mesogens 1,4di-[4-(6-acryloyloxyhexyloxy)benzoyloxy]-2-methylbenzene (**1**), 4-methoxyphenyl 4-(6-(acryloyloxy)hexyloxy)benzoate (**2**), and 4-cyanophenyl 4-((6-(acryloyloxy)hexyl) oxy)benzoate (**3**) were obtained from Merck UK. Disperse red 1 (**4**) and butylated hydroxytoluene (**6**) were received from Sigma-Aldrich. Irgacure 819 (**5**) was purchased from Ciba. Dichloromethane (DCM) was obtained from Sigma-Aldrich. Polyimide SE-5661 was supplied by Nissan Chemical Industries. Optimer AL 1254 was purchased from JSR. All chemicals were used as received.

### Preparation of autonomous snappers

A typical recipe used for the fabrication of LCN contained 24.5 wt % monomer **1**, 49 wt % monomer **2**, 24.5 wt % monomer **3**, 0.5 wt % photosensitive dye **4**, 1 wt % photoinitiator **5**, and 0.5 wt % inhibitor **6**. The concentration of dye **4** was chosen to provide an adequate photothermal heating rate while maintaining a uniform temperature distribution across the film. Recipes used for the fabrication of L-LCN and D-LCN contained 9.75 wt % monomer **1**, 87.75 wt % monomer **2**, and 97.5 wt % monomer **1**, respectively. Mixtures for L-LCN and D-LCN did not contain monomer **3**, while they had the same concentration of photosensitive dye **4**, photoinitiator **5**, and inhibitor **6**. Components were dissolved in DCM to achieve homogeneous mixing. DCM was then evaporated at 30°C under argon flow. Glass substrates were cleaned with acetone and isopropanol in an ultrasonic bath for 20 min, dried with blown air, and placed in an ultraviolet (UV)–ozone photoreactor for 15 min. After the ozone reaction, glass substrates were spin coated with 5661 polyimide and Optimer AL 1254 to induce homeotropic and planar alignment, respectively. Coated glass substrates were prebaked at 110°C for 10 min on a hotplate and cured at 180°C for 1.5 hours in an oven. Substrates coated with AL 1254 were rubbed on a cloth in one direction. To achieve splay-bend alignment, an LC monomer mixture was introduced between glass substrates treated with 5661 polyimide and Optimer AL 1245. Consequently, the molecules align parallel to the surface on the side pretreated with AL 1254 and perpendicular to the surface on the side pretreated with 5661 polyimide. Between the two surfaces, the molecular orientation transitions gradually from planar to perpendicular, driven by the elastic properties of the LC molecules. Substrates were glued to each other with 20-μm spacers to fix the thickness of the LCN. The LC mixture was filled into the cells at 80°C (110°C for D-LCN). Then, the cells were cooled down to 30°C (47°C for L-LCN and 90°C for D-LCN) and photopolymerized through a photomask with a light intensity of 30 mW/cm^2^ for 12 s (20 s for L-LCN and 8 s for D-LCN). Samples were then photopolymerized at 100°C (130°C for D-LCN) without a photomask for 10 min. Photopolymerization conditions were chosen according to the transition temperatures of LC mixtures to ensure the nematic phase in the first step and the isotropic phase in the second step of photopolymerization (fig. S14). LCNs were then postbaked at 120°C for 20 min to release the stress induced by shrinkage due to polymerization. Freestanding LCNs were removed from the cell using a razor blade.

### Characterization

Transition temperatures of LC mixtures were analyzed with differential scanning calorimetry (DSC Q1000, TA Instruments) equipped with a cooling accessory. Three cycles from −50° to 150°C at a rate of 10°C/min were performed. The results of the third cycle were used for the analysis. The mesophases were verified using a polarized optical microscope (CTR6000, Leica) equipped with a Leica DFC 420C camera. The temperature was controlled using a Linkam THMS600 heating and cooling unit. Glass transition temperatures ($T_{g}$) of LCNs were determined with dynamic mechanical analysis (Q800 DMA, TA Instruments). The temperature was ramped from 35° to 150°C using 20-μm amplitude at a frequency of 1 Hz. $T_{g}$ values were obtained from tan(δ) peaks. A tensile test of LCNs was performed on splay-aligned samples in a parallel orientation relative to the nematic director at 25°C using a force ramp of 3 N/min. Young’s modulus values of LCNs were calculated from the linear region of the stress-strain curves. The absorption spectrum of the LCN was obtained using a UV/Visible/Near Infrared spectrometer (Lambda 950, Perkin Elmer) equipped with an integrating sphere detector. Surface profiles of TMLs with different surface roughness were obtained with the DektakXT Stylus Profiler. LCNs were placed planar side up facing the 455-nm light-emitting diode (LED) light (M455L4, Thorlabs) equipped with a Kohler Illumination setup. Typically, LED light was placed 17 cm above the LCN and had a full width at half maximum of 12 mm. Light intensity was calculated from the calibration data obtained with a UV radiometer (Opsytec Dr. Grobel) (fig. S15). Actuation videos were recorded with Olympus digital camera at 30 or 60 fps. Infrared (IR) videos were recorded with Xenics Gobi high-speed IR camera (30 to 50 fps). Video analysis was performed with Blender. The center of mass of active segments was tracked with a custom script to calculate their displacements. The images were analyzed without any modification. The frequency of self-sustained snapping was obtained by fast Fourier transformation of smooth data.

### Simulation of the deformation of LCNs

In our computations, we model an LCN as a 2D concave-shaped ribbon, drawing inspiration from prior research on deformable structures in lattice Boltzmann fluids (*55*, *56*). We use a bead-spring model to simulate the LCN, where each node *N_i_* is linked to its neighboring nodes $N_{i+1}$ and $N_{i-1}$. The energy of the LCN comprises two main components

Emembrane=Eelastic+Ebending

Elastic energy (stretching): This accounts for surface stretch and is represented by the elastic energy term ($E^{\text{elastic}}$). It is calculated as the sum over neighboring nodes, with $K_{\text{str}}$ denoting the elastic coefficient for stretching.

$$E^{\text{elastic}}=\sum_{\text{neighbor}\left(i,j\right)}\frac{1}{2}K_{\text{str}}{\left(r_{\mathrm{ij}}-r_{\mathrm{ij}}^{0}\right)}^{2}$$

Bending energy: This arises from surface curvature and is expressed as a sum over all nodes, with $K_{\text{curv}}$ representing the coefficient penalizing LCN bending.

$$E^{\text{bending}}=\sum \frac{1}{2}K_{\text{curv}}{\left(\theta_{i}-\pi \right)}^{2}$$

The free energy of LC networks is described in terms of the Q tensor. The total free energy (F) of the LC includes terms representing the nematic order, along with elastic constants ($L_{1}$) and Landau parameters (A and U). In addition, a surface anchoring energy term ($f_{S}$) is introduced to ensure specific anchoring of the LC molecules at surfaces (*51*, *57*)

$$f_{\text{LdG}}=\frac{A}{2}\left(1-\frac{U}{3}\right)\text{Tr}\left(Q^{2}\right)-\frac{\mathrm{AU}}{3}\text{Tr}\left(Q^{3}\right)+\frac{\mathrm{AU}}{4}{\left[\text{Tr}\left(Q^{2}\right)\right]}^{2}$$

$$f_{E}=\frac{1}{2}L_{1}{\left(\nabla Q\right)}^{2}$$

$$f_{S}=\frac{1}{2}W{\left(Q-Q^{s}\right)}^{2}$$

We use the Euler-Lagrange equation to minimize the free energy numerically (*54*). The equation of motion for each bead $\left(N_{i,j}\right)$ considers external force (LC force), friction, and velocity. Forces acting on the LCN nodes include surface tension effects and LC-induced stress. For detailed information on the calculation of the LC force, please refer to the cited paper (*52*).

$$m\ddot{r}=-\frac{\partial E^{\text{membrane}}}{\partial r_{i}}+F_{i}^{\text{ext}}-\gamma v_{i}$$

Within the bulk nematic of the central region, the gradient of orientation in the splay-bend director field gives rise to forces F.

$$F=\frac{\partial E}{\partial z}$$

$$E=f-\frac{2W}{K}f_{s}\left(Q\right)$$

$$f=f_{\text{Ld}}+f_{E}$$

In our simulations, we make several assumptions regarding the dimensions and properties of LCNs. The total length of the LCNs is set to 528 μm, with a thickness of 10 μm between the upper and lower surfaces. The initial bent LCNs span a horizontal distance of 500 μm between their left and right ends. The top end of the LCNs is positioned 70 μm away from the metal substrate. In addition, we specify Landau coefficients: $A=10^{4}$ N m^−2^ K^−1^, and U=6 K for the N state, while for the Iso state, U=8/3 K. We assume strong anchoring for both the upper and lower surfaces; hence, $f_{S}\left(Q\right)=0$. These parameters provide the foundation for our simulations and help establish the conditions under which we investigate the behavior of LCNs.

## References

[1] B. Rosslenbroich, History, Philosophy and Theory of the Life Sciences, *On the Origin of Autonomy* (Springer, Cham, ed. 1, 2014), vol. 5.

[2] A. Walther, Viewpoint: From responsive to adaptive and interactive materials and materials systems: A roadmap. Adv. Mater. 32, 1905111 (2020). 10.1002/adma.201905111 PMC7612550 31762134

[3] M. M. Lerch, A. Grinthal, J. Aizenberg, Viewpoint: Homeostasis as inspiration—Toward interactive materials. Adv. Mater. 32, 1905554 (2020). 10.1002/adma.201905554 31922621

[4] R. Merindol, A. Walther, Materials learning from life: Concepts for active, adaptive and autonomous molecular systems. Chem. Soc. Rev. 46, 5588–5619 (2017). 28134366 10.1039/c6cs00738d

[5] F. Lancia, A. Ryabchun, N. Katsonis, Life-like motion driven by artificial molecular machines. Nat. Rev. Chem. 3, 536–551 (2019).

[6] C. Kaspar, B. J. Ravoo, W. G. van der Wiel, S. V. Wegner, W. H. P. Pernice, The rise of intelligent matter. Nature 594, 345–355 (2021). 34135518 10.1038/s41586-021-03453-y

[7] S. Zhou, Y. Li, Q. Wang, Z. Lyu, Integrated actuation and sensing: Toward intelligent soft robots. Cyborg Bionic Syst. 5, 0105 (2024). 38711958 10.34133/cbsystems.0105 PMC11070852

[8] Y. Cao, M. Derakhshani, Y. Fang, G. Huang, C. Cao, Bistable structures for advanced functional systems. Adv. Funct. Mater. 31, 2106231 (2021).

[9] Y. Chi, Y. Li, Y. Zhao, Y. Hong, Y. Tang, J. Yin, Bistable and multistable actuators for soft robots: Structures, materials, and functionalities. Adv. Mater. 34, 2110384 (2022). 10.1002/adma.202110384 35172026

[10] L. Chen, K. Tan, S. Yang, Q. Deng, Evoking the snap-through instability in hard-magnetic soft materials: Rapid actuation and giant deformation. Int. J. Solids Struct. 246-247, 111607 (2022).

[11] Y. Luo, D. K. Patel, Z. Li, Y. Hu, H. Luo, L. Yao, C. Majidi, Intrinsically multistable soft actuator driven by mixed-mode snap-through instabilities. Adv. Sci. 11, 2307391 (2024). 10.1002/advs.202307391 PMC11095224 38447200

[12] M. L. Smith, G. M. Yanega, A. Ruina, Elastic instability model of rapid beak closure in hummingbirds. J. Theor. Biol. 282, 41–51 (2011). 21609721 10.1016/j.jtbi.2011.05.007

[13] Y. Forterre, J. M. Skotheim, J. Dumals, L. Mahadevan, How the Venus flytrap snaps. Nature 433, 421–425 (2005). 15674293 10.1038/nature03185

[14] J. Jeon, J.-C. Choi, H. Lee, W. Cho, K. Lee, J. G. Kim, J.-W. Lee, K.-I. Joo, M. Cho, H.-R. Kim, J. J. Wie, Continuous and programmable photomechanical jumping of polymer monoliths. Mater. Today 49, 97–106 (2021).

[15] T. S. Hebner, K. Korner, C. N. Bowman, K. Bhattacharya, T. J. White, Leaping liquid crystal elastomers. Sci. Adv. 9, eade1320 (2023). 36652507 10.1126/sciadv.ade1320 PMC9848472

[16] H. Lee, C. Xia, N. X. Fang, First jump of microgel; actuation speed enhancement by elastic instability. Soft Matter 6, 4342–4345 (2010).

[17] B. Gorissen, D. Melancon, N. Vasios, M. Torbati, K. Bertoldi, Inflatable soft jumper inspired by shell snapping. Sci. Robot. 5, eabb1967 (2020). 33022625 10.1126/scirobotics.abb1967

[18] X. Zhou, G. Chen, B. Jin, H. Feng, Z. Chen, M. Fang, B. Yang, R. Xiao, T. Xie, N. Zheng, Multimodal autonomous locomotion of liquid crystal elastomer soft robot. Adv. Sci. 11, 2402358 (2024). 10.1002/advs.202402358 PMC11187929 38520731

[19] Y. Wanga, Q. Wang, M. Liu, Y. Qin, L. Cheng, O. Bolmin, M. Alleyne, A. Wissa, R. H. Baughman, D. Vella, S. Tawfick, Insect-scale jumping robots enabled by a dynamic buckling cascade. Proc. Natl. Acad. Sci. U.S.A. 120, e2210651120 (2017). 10.1073/pnas.2210651120 PMC9945960 36689664

[20] Y. Chi, Y. Hong, Y. Zhao, Y. Li, J. Yin, Snapping for high-speed and high-efficient butterfly stroke–like soft swimmer. Sci. Adv. 8, eadd3788 (2022). 36399554 10.1126/sciadv.add3788 PMC9674291

[21] Y. Tang, Y. Chi, J. Sun, T. H. Huang, O. H. Maghsoudi, A. Spence, J. Zhao, H. Su, J. Yin, Leveraging elastic instabilities for amplified performance: Spine-inspired high-speed and high-force soft robots. Sci. Adv. 6, eaaz6912 (2020). 32494714 10.1126/sciadv.aaz6912 PMC7209986

[22] Y. Chi, Y. Tang, H. Liu, J. Yin, Leveraging monostable and bistable pre-curved bilayer actuators for high-performance multitask soft robots. Adv. Mater. Technol. 5, 2000370 (2020).

[23] J. Gao, A. Clement, M. Tabrizi, M. R. Shankar, Molecularly directed, geometrically latched, impulsive actuation powers sub-gram scale motility. Adv. Mater. Technol. 7, 2100979 (2022).

[24] D. S. Kim, Y. J. Lee, Y. B. Kim, Y. Wang, S. Yang, Autonomous, untethered gait-like synchronization of lobed loops made from liquid crystal elastomer fibers via spontaneous snap-through. Sci. Adv. 9, eadh5107 (2023). 37196078 10.1126/sciadv.adh5107 PMC10191433

[25] Y. Zhao, Y. Hong, Y. Li, F. Qi, H. Qing, H. Su, J. Yin, Physically intelligent autonomous soft robotic maze escaper. Sci. Adv. 9, eadi3254 (2023). 37682998 10.1126/sciadv.adi3254 PMC10491293

[26] S. Poppinga, C. Weisskopf, A. S. Westermeier, T. Masselter, T. Speck, Fastest predators in the plant kingdom: Functional morphology and biomechanics of suction traps found in the largest genus of carnivorous plants. AoB Plants 8, plv140 (2016). 10.1093/aobpla/plv140 PMC4717191 26602984

[27] M. Ravi Shankar, M. L. Smith, V. P. Tondiglia, K. M. Lee, M. E. McConney, D. H. Wang, L.-S. Tan, T. J. White, Contactless, photoinitiated snap-through in azobenzene-functionalized polymers. Proc. Natl. Acad. Sci. U.S.A. 110, 18792–18797 (2013). 24190994 10.1073/pnas.1313195110 PMC3839691

[28] Q. Zhao, X. Yang, C. Ma, D. Chen, H. Bai, T. Li, W. Yang, T. Xie, A bioinspired reversible snapping hydrogel assembly. Mater. Horizons 3, 422–428 (2016).

[29] D. J. Preston, H. J. Jiang, V. Sanchez, P. Rothemund, J. Rawson, M. P. Nemitz, W.-K. Lee, Z. Suo, C. J. Walsh, G. M. Whitesides, A soft ring oscillator. Sci. Robot. 4, eaaw5496 (2019). 33137768 10.1126/scirobotics.aaw5496

[30] X. Zhang, Y. Wang, Z. Tian, M. Samri, K. Moh, R. M. McMeeking, R. Hensel, E. Arzt, A bioinspired snap-through metastructure for manipulating micro-objects. Sci. Adv. 8, eadd4768 (2022). 36399572 10.1126/sciadv.add4768 PMC9674295

[31] Y. Kim, J. van den Berg, A. J. Crosby, Autonomous snapping and jumping polymer gels. Nat. Mater. 20, 1695–1701 (2021). 33526877 10.1038/s41563-020-00909-w

[32] Z.-Z. Nie, M. Wang, H. Yang, Self-sustainable autonomous soft actuators. Commun. Chem. 7, 58 (2024). 38503863 10.1038/s42004-024-01142-1 PMC10951225

[33] Y. Zhao, Y. Hong, F. Qi, Y. Chi, H. Su, J. Yin, Self-sustained snapping drives autonomous dancing and motion in free-standing wavy rings. Adv. Mater. 35, 2207372 (2023). 10.1002/adma.202207372 36366927

[34] H. Seung Choi, D. Seok Kim, Self-repeatable snapping liquid–crystal-elastomer actuator. Chem. Eng. J. 500, 156744 (2024).

[35] A. H. Gelebart, D. Jan Mulder, M. Varga, A. Konya, G. Vantomme, E. W. Meijer, R. L. B. Selinger, D. J. Broer, Making waves in a photoactive polymer film. Nature 546, 632–636 (2017). 28658225 10.1038/nature22987 PMC5495175

[36] Z. Deng, H. Zhang, A. Priimagi, H. Zeng, Light-fueled nonreciprocal self-oscillators for fluidic transportation and coupling. Adv. Mater. 36, 2209683 (2024). 10.1002/adma.202209683 36525600

[37] Y. Geng, R. Kizhakidathazhath, J. P. F. Lagerwall, Robust cholesteric liquid crystal elastomer fibres for mechanochromic textiles. Nat. Mater. 21, 1441–1447 (2022). 36175519 10.1038/s41563-022-01355-6 PMC9712110

[38] Y. Chen, C. Valenzuela, X. Zhang, X. Yang, L. Wang, W. Feng, Light-driven dandelion-inspired microfliers. Nat. Commun. 14, 3036 (2023). 37236989 10.1038/s41467-023-38792-z PMC10219969

[39] O. M. Wani, H. Zeng, A. Priimagi, A light-driven artificial flytrap. Nat. Commun. 8, 15546 (2017). 28534872 10.1038/ncomms15546 PMC5457518

[40] Y. You, Y. M. Golestani, D. J. Broer, T. Yang, G. Zhou, R. L. B. Selinger, D. Yuan, D. Liu, Transforming patterned defects into dynamic poly-regional topographies in liquid crystal oligomers. Mater. Horizons 11, 3178–3186 (2024). 10.1039/d4mh00131a PMC11216033 38666445

[41] H. Shahsavan, A. Aghakhani, H. Zeng, Y. Guo, Z. S. Davidson, A. Priimagi, M. Sitti, Bioinspired underwater locomotion of light-driven liquid crystal gels. Proc. Natl. Acad. Sci. U.S.A. 117, 5125–5133 (2020). 32094173 10.1073/pnas.1917952117 PMC7071923

[42] P. Sartori, R. S. Yadav, J. del Barrio, A. DeSimone, C. Sánchez-Somolinos, Photochemically induced propulsion of a 4D printed liquid crystal elastomer biomimetic swimmer. Adv. Sci. 11, 2308561 (2024). 10.1002/advs.202308561 PMC11220691 38590131

[43] J.-A. Lv, Y. Liu, J. Wei, E. Chen, L. Qin, Y. Yu, Photocontrol of fluid slugs in liquid crystal polymer microactuators. Nature 537, 179–184 (2016). 27604946 10.1038/nature19344

[44] Z. Yang, J. Li, X. Chen, Y. Fan, J. Huang, H. Yu, S. Yang, E.-Q. Chen, Precisely controllable artificial muscle with continuous morphing based on “breathing” of supramolecular columns. Adv. Mater. 35, 2211648 (2023). 10.1002/adma.202211648 36634260

[45] L. C. van Laake, J. T. B. Overvelde, Bio-inspired autonomy in soft robots. Commun. Mater. 5, 198 (2024).

[46] M. Sitti, Physical intelligence as a new paradigm. Extrem. Mech. Lett. 46, 101340 (2021). PMC7612657 35475112

[47] W. Feng, Q. He, L. Zhang, Embedded physical intelligence in liquid crystalline polymer actuators and robots. Adv. Mater. 37, 2312313 (2024). 38375751 10.1002/adma.202312313 PMC11733722

[48] D. S. Polat, M. Zmyślony, J. S. Biggins, D. Liu, Spontaneous snap-through of strongly buckled liquid crystalline networks. Extrem. Mech. Lett. 68, 102149 (2024).

[49] S. Armon, E. Efrati, R. Kupferman, E. Sharon, Geometry and mechanics in the opening of chiral seed pods. Science 333, 1726–1730 (2010). 10.1126/science.1203874 21940888

[50] M. Gomez, D. E. Moulton, D. Vella, Critical slowing down in purely elastic “snap-through” instabilities. Nat. Phys. 13, 142–145 (2017).

[51] P. G. De Gennes, J. Prost, *The Physics of Liquid Crystals* (Oxford Univ. Press, 1993); 10.1093/oso/9780198520245.001.0001.

[52] R. Zhang, Y. Zhou, J. A. Martínez-González, J. P. Hernández-Ortiz, N. L. Abbott, J. J. De Pablo, Controlled deformation of vesicles by flexible structured media. Sci. Adv. 2, e1600978 (2016). 27532056 10.1126/sciadv.1600978 PMC4980106

[53] F. E. MacKay, C. Denniston, Deformable vesicles interacting in a nematic liquid crystal. Soft Matter 9, 5285–5292 (2013).

[54] M. Ravnik, S. Žumer, Landau-de Gennes modelling of nematic liquid crystal colloids. Liq. Cryst. 36, 1201–1214 (2009).

[55] M. M. Dupin, I. Halliday, C. M. Care, L. Alboul, L. L. Munn, Modeling the flow of dense suspensions of deformable particles in three dimensions. Phys. Rev. E 75, 066707 (2007). 10.1103/PhysRevE.75.066707 PMC2752716 17677389

[56] G. A. Buxton, R. Verberg, D. Jasnow, A. C. Balazs, Newtonian fluid meets an elastic solid: Coupling lattice Boltzmann and lattice-spring models. Phys. Rev. E 71, 056707 (2005). 10.1103/PhysRevE.71.056707 16089691

[57] A. G. Kalugin, Surface waves in nematic liquid crystals. Moscow Univ. Mech. Bull. 67, 76–77 (2012).

