# Supplementary material for: An autonomous snapper featuring adaptive actuation and embodied intelligence
Source: Sci Adv. 2025 Apr 4;11(14):eadu4268. doi: 10.1126/sciadv.adu4268 (PMC11970476; doi:10.1126/sciadv.adu4268)
Supplement: Supplementary file 1 — Figs. S1 to S17 Table S1 Legends for movies S1 to S5 [file sciadv.adu4268_sm.pdf]

Science Advances
AAAS

Supplementary Materials for

An autonomous snapper featuring adaptive actuation and embodied intelligence

Duygu S. Polat *et al.*

Corresponding author: Danqing Liu, d.liu1@tue.nl; Satoshi Aya, satoshiaya@scut.edu.cn

Sci. Adv. **11**, eadu4268 (2025)
DOI: 10.1126/sciadv.adu4268

The PDF file includes:

Figs. S1 to S17
Table S1
Legends for movies S1 to S5

Other Supplementary Material for this manuscript includes the following:

Movies S1 to S5

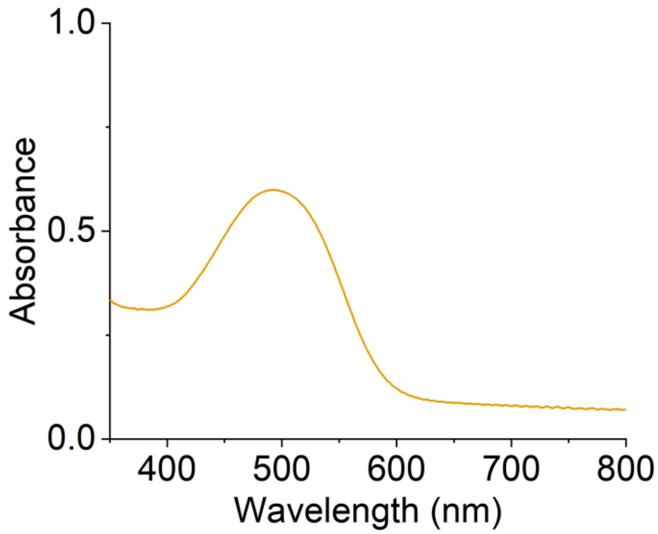

Fig. S1.

Absorption spectra of the LCN fabricated with photothermal dye 4.

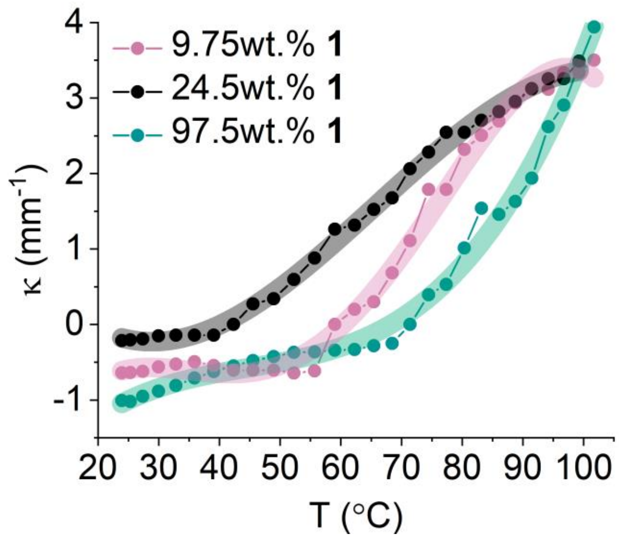

Fig. S2.

The thermal bending of hybrid LCNs with different monomer 1 composition exposed to homogenous heat in an oven. Y-axis correspond to the bending curvature (κ) of the LCNs

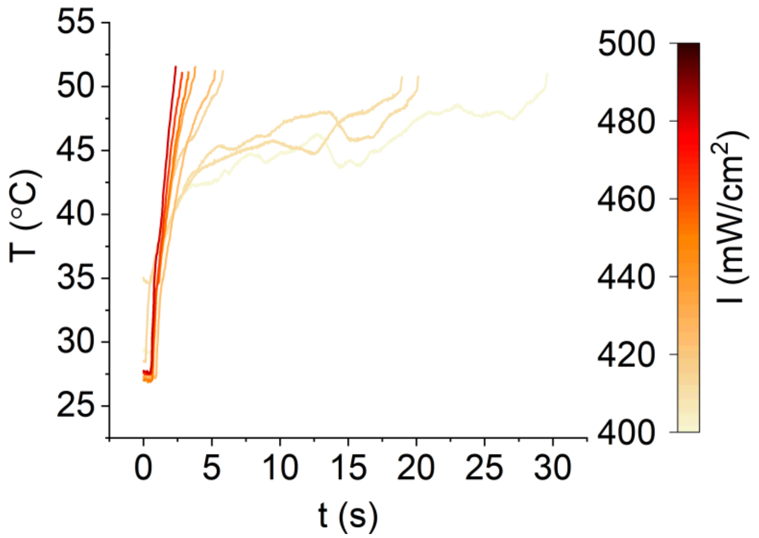

Fig. S3.

Light intensity dependency of snap-through. Temperature evolution of the LCN prior to snap-through upon illumination with different light intensities.

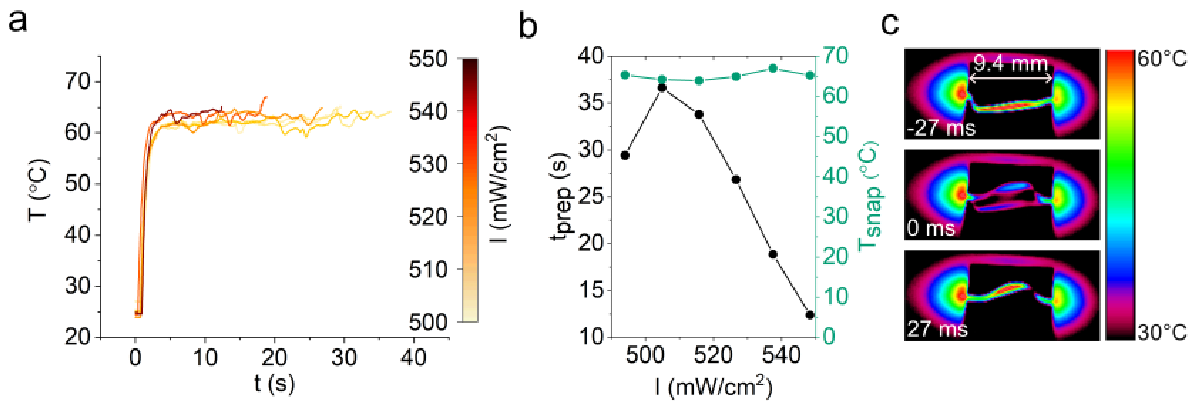

Fig. S4.

Light intensity dependency of snap-through for snappers with different initial compression (L/L_0). A. Temperature evolution of snapper with 0.94 initial compression. B. Influence of light intensity on the preparation time and temperature of its snap-through instability. C. IR images of the LCN during snap-through.

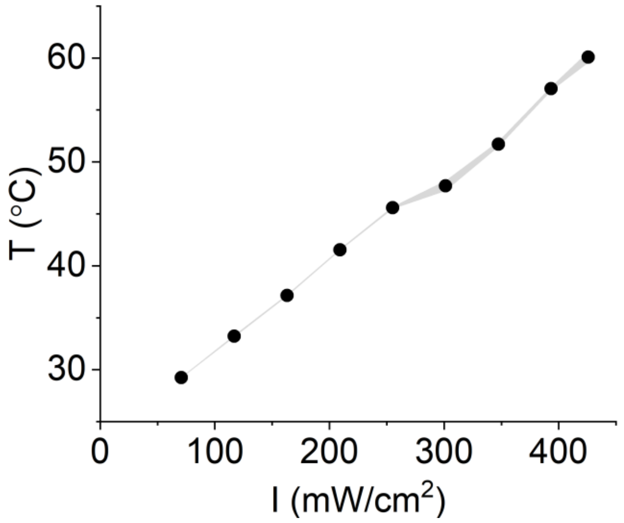

Fig. S5.

Relationship between light intensity and temperature of LCN with 24.5 wt.% monomer 1. For light intensities below 400 mW/cm^2, LCN does not exhibit self-snapping. Between 250 and 400 mW/cm^2 small amplitude temperature oscillations that do not lead to snap-through can be observed as signified by standard deviation of temperature recordings.

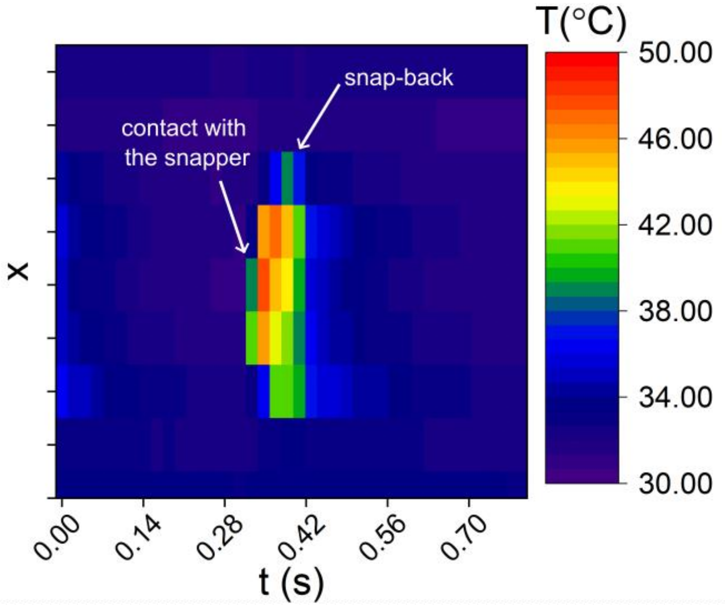

Fig S6.

Temperature profile of TML shadowed during self-sustained snapping. After snap back it takes 0.14 s for TML to return to its initial temperature profile before contact with snapper.

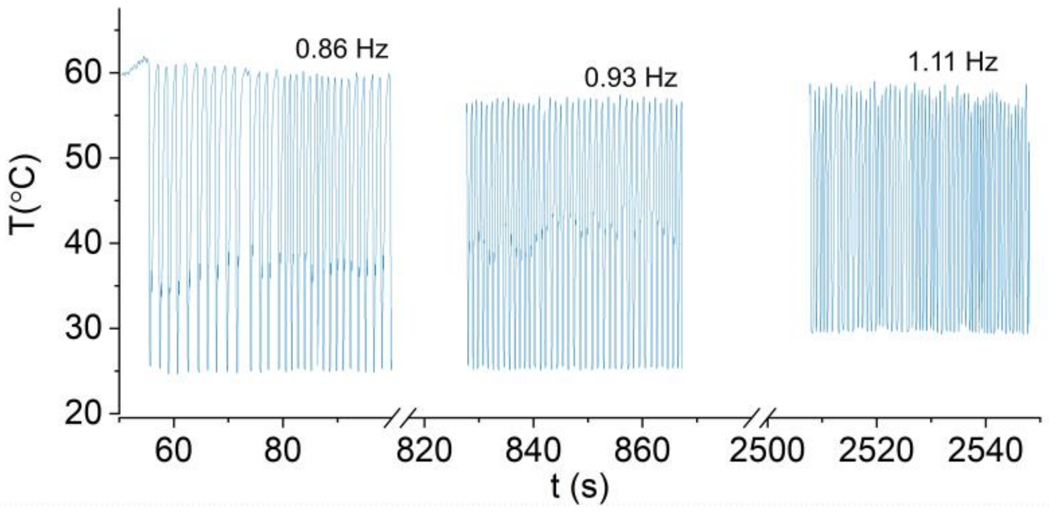

Fig. S7.

Long term performance of the autonomous snapper under illumination with light intensity of 450 mW/cm^2.

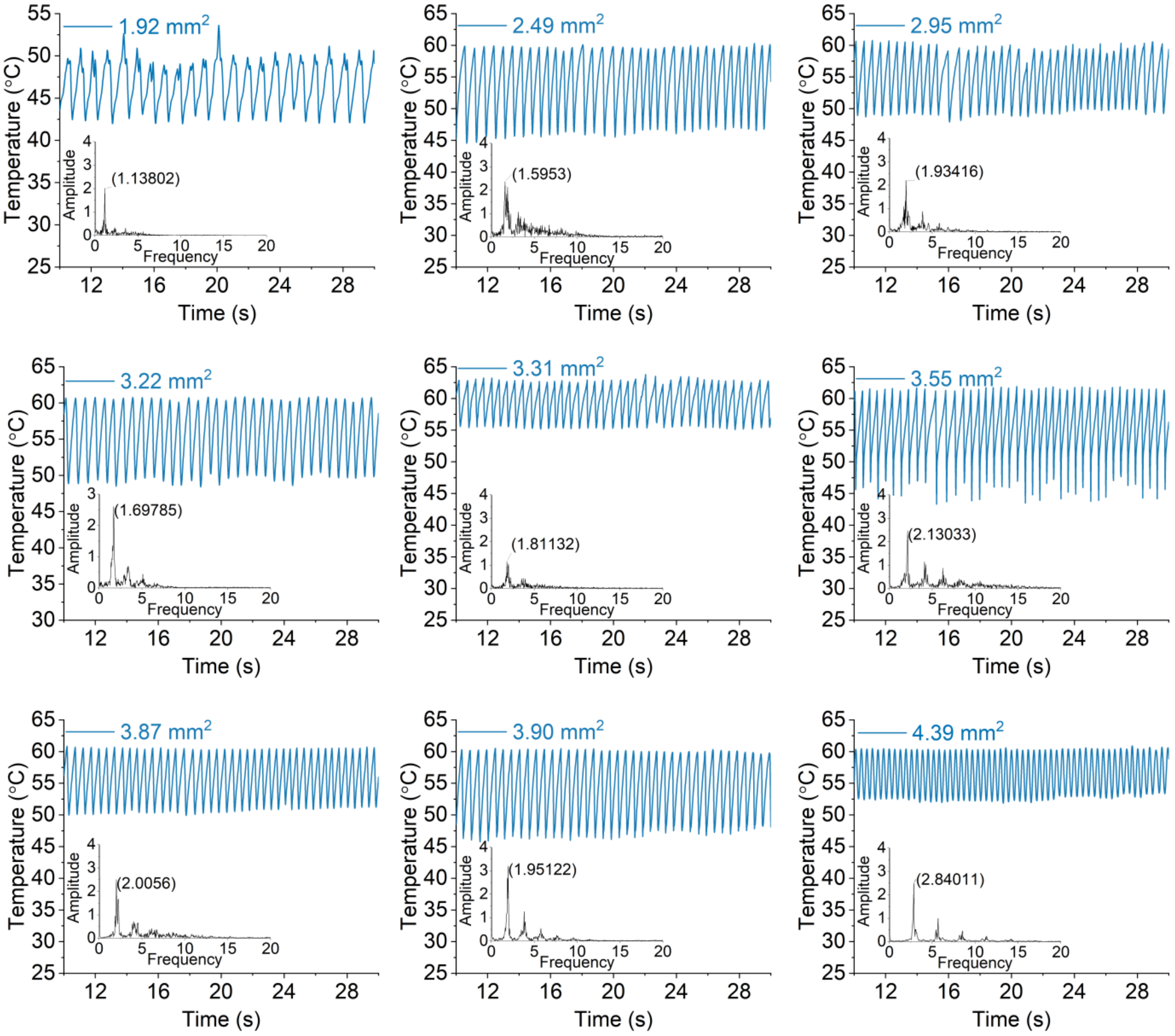

Fig. S8.

Heat transfer area dependency of self-sustained snapping. Temperature oscillations and corresponding FFT plots for self-sustained snapping at different heat transfer area.

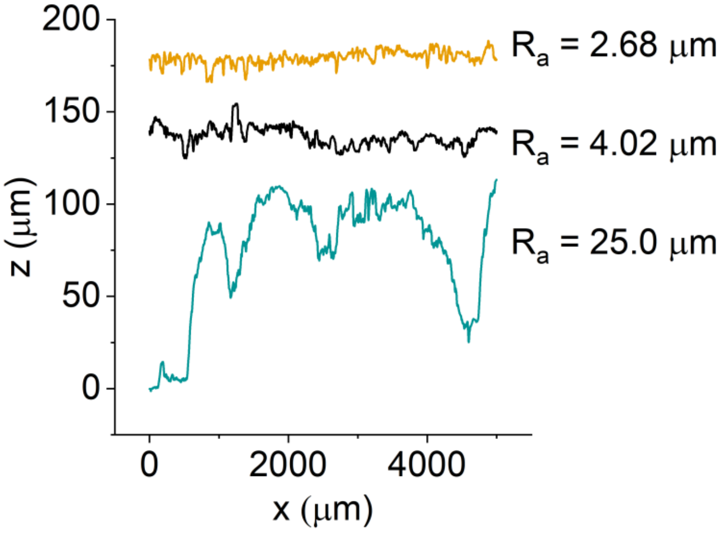

Fig. S9.

Surface profiles of TMLs with varying roughness average (R_a).

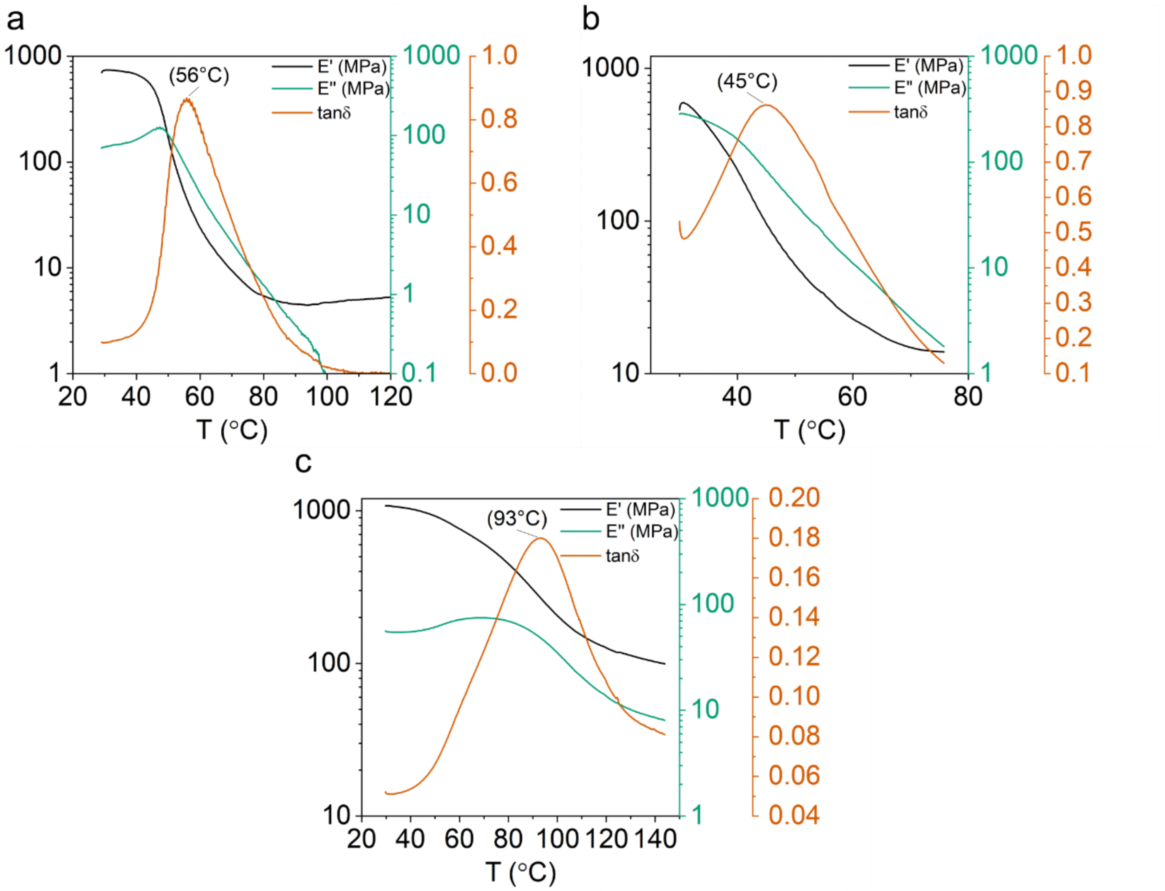

Fig. S10.

Mechanical properties of hybrid LCNs. DMTA measurement results for LCNs fabricated with (a) 24.5wt.%, (b) 9.75wt.% (L-LCN) and (c) 97.5wt.% (D-LCN) monomer 1.

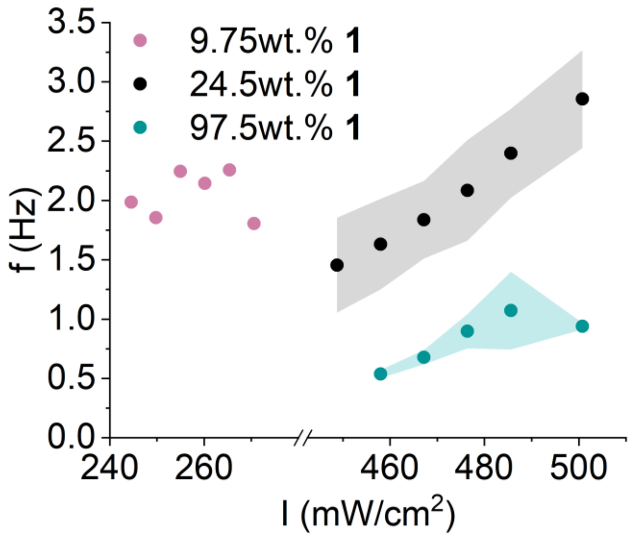

Fig. S11.

Influence of light intensity on the frequency of self-snapping for LCN strips with different diacrylate content. LCNs with 24.5 wt.% and 97.5 wt.% of monomer 1 do not exhibit self-sustained snapping at light intensities below 400 and 460 mW/cm², respectively. However, above these threshold light intensities, their oscillation frequencies display a linear relationship with light intensity. When the light intensity exceeds 500 mW/cm², the photothermal heating rate of the LCN surpasses the heat transfer rate to TML, resulting in the end of self-sustained snapping. In contrast, the LCN with 9.75 wt.% of monomer 1 demonstrates self-sustained snapping within a light intensity range of 245 to 275 mW/cm², with its oscillation frequency remaining independent of light intensity. This narrow operational light intensity range is because 275 mW/cm² is sufficient to keep the polymer network well above its glass transition temperature (T_g). In the rubbery state due to the influence of gravity, the LCN cannot resist deformation, which is essential for snap-through instability.

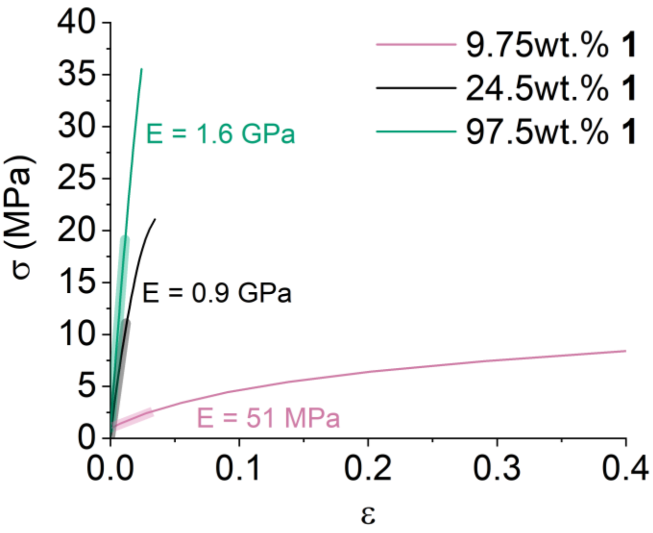

Fig. S12.

Stress-strain curves for LCNs measured parallel to the nematic director at 25°(C) Bending stiffness (B) can be calculated using the formula B= $(Et^3)/12$ where t is the thickness and 0.02 mm for all the samples.

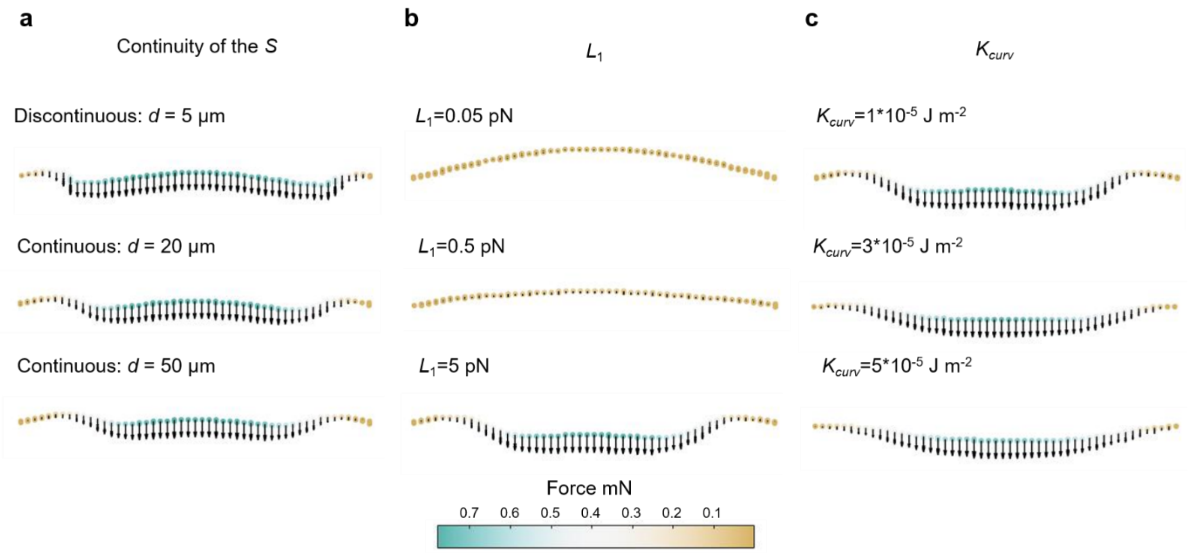

Fig. S13.

Deformation process of snapper under different parameters. (a) This column demonstrates how the continuity of the order parameter in the transition region affects the deformation process of snapper. The three images record the force field distribution at t = 60 μs. A comparison reveals that when the order parameter in the transition region is discontinuous, the forces exerted on the transition region are greater than those in the case of continuous transition, leading to the snapper with discontinuous transition touching the bottom earlier compared to the N phase. (b) This column shows the significant influence of liquid crystal elastic parameters on the deformation process of snapper. The analysis results indicate that liquid crystal elasticity is a crucial factor driving the downward movement of the N phase region in snapper. Specifically, a larger liquid crystal elastic parameter implies stronger interactions between liquid crystal molecules, leading to greater energy accumulation and more pronounced deformation effects. (c) This column explores the influence of the bending coefficient on the deformation process of snapper. Through simulation, it is found that the bending coefficient directly controls the curvature of snapper. Specifically, as the bending coefficient (K_{curv}) increases, the curvature of snapper decreases, making the connection between the iso phase and N phase smoother.

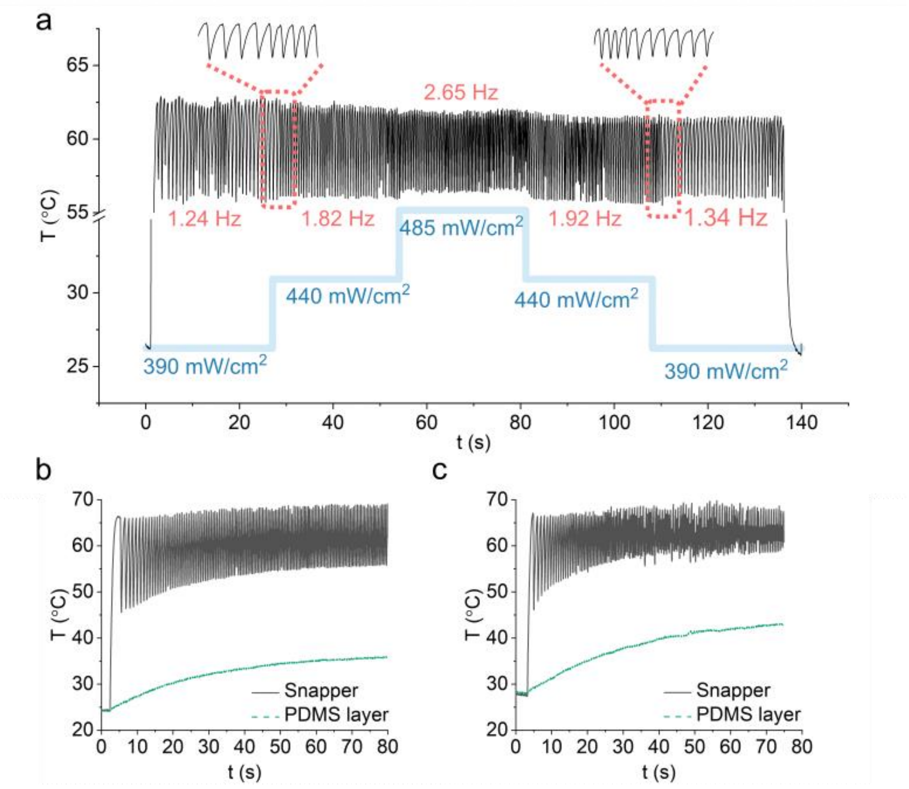

Fig. S14.

Latency and adaptability of autonomous snapper (a) Temperature oscillations to step-wise changes in light intensity for 20 seconds waiting time (b) Temperature oscillations to increasing surface temperature with a rate of 0.3°C/min (c) Temperature oscillations to increasing surface temperature with a rate of 0.4°C/min.

As the mechanism of self-sustained snapping relies on the energy transfer, snapper is quite responsive to changing conditions in the environment. We wanted to investigate how robust self-snapping can be after the removal of these changes, and how fast it can go back to the original state. **Fig. S14A** shows the response of the snapper to step increases and decreases in light intensity. We altered the intensity of the light between 390 and 485 mW/cm^2 in two steps and allowed the snapper 20 seconds in between to respond. Upon increasing and decreasing the light intensity, the snapper rapidly accelerated and slowed down, respectively. During the step decrease periods to 440 and 390 mW/cm^2, frequency of oscillations increased by 9% and 4.5% respectively compared to step increase periods. This could be explained by the influence of material history on the frequency of oscillations and the slow rate of convective heat transfer to the surroundings.

Fig. S14B and **Fig. S14C** show the response of the snapper to changes in T_S. For this purpose, we chose polydimethylsiloxane (PDMS) as TML due to its low thermal conductivity. We observed that the LCN adapts to the changes in $T_S$by adjusting its frequency when the photothermal heating rate of TML was 0.3°C/min. As T_S increases, temperature gradient between the snapper and TML remains the same (24°C) while the temperature difference of the LCN

between state 3 and state 1 decreases from 20°C to 14°C, and the frequency of oscillations increases by 5%. However, when the photothermal heating rate of TML exceeds 0.4°C/min, snapper starts to exhibit unstable oscillations which can be seen in **Fig. S13C**, which could also be explained by the rubbery state of both the snapper and PDMS at this temperature (42°C) causing adhesion between interfaces (**Fig. S15**).

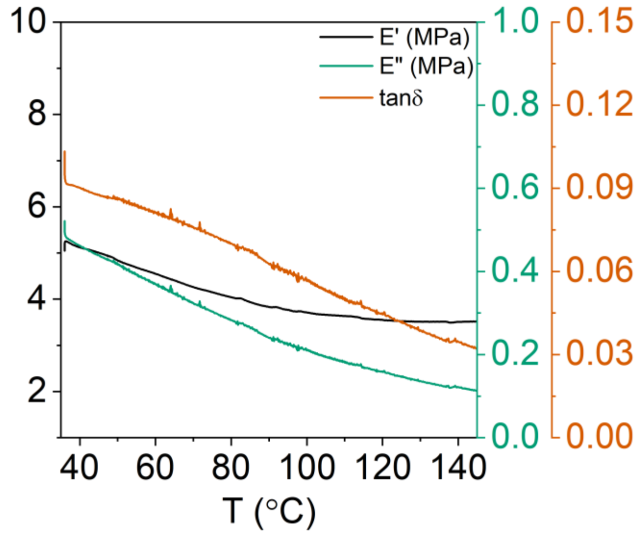

Fig. S15.
DMTA measurement results for TML designed with PDMS.

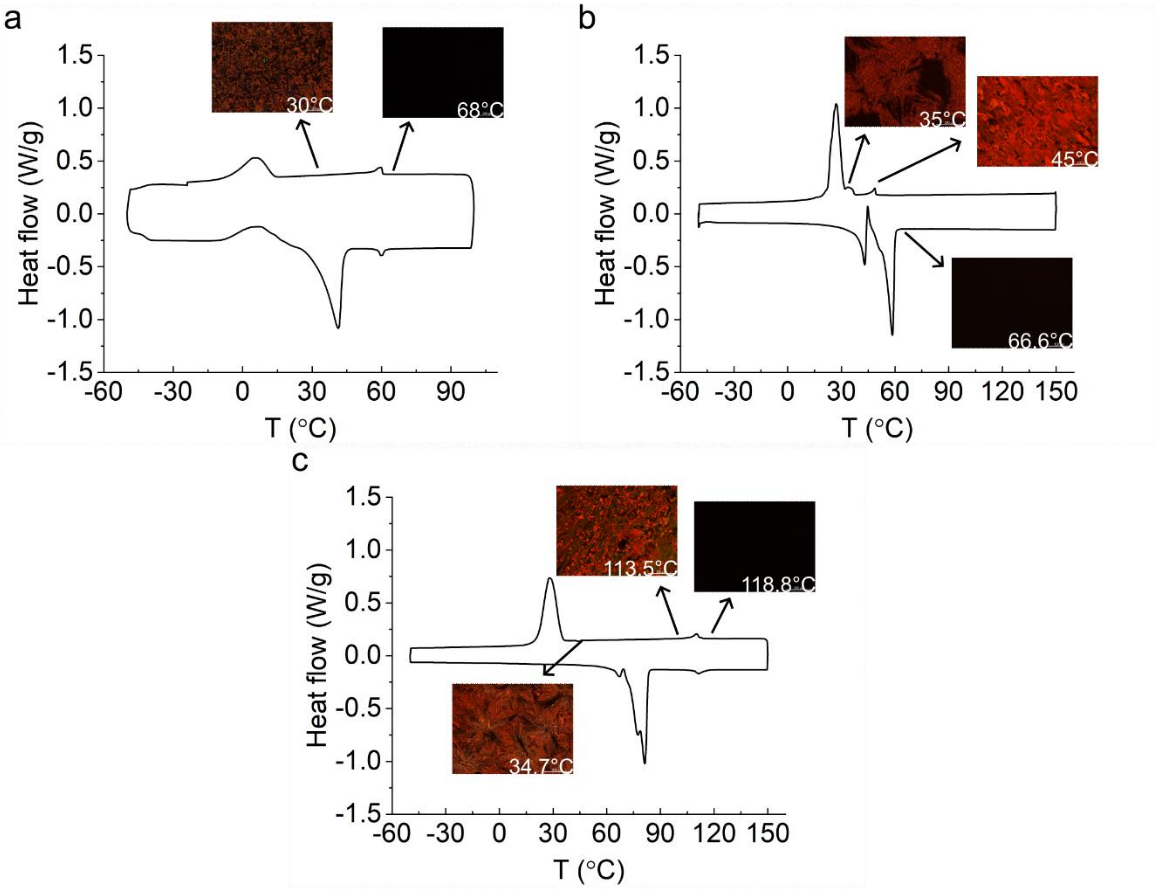

Fig. S16.

Liquid crystal phase transitions. DSC measurement results for LC mixtures with (A) 24.5wt.%, (B) 9.75wt.% and (C) 97.5wt.% monomer 1 and POM images depicting phase transitions.

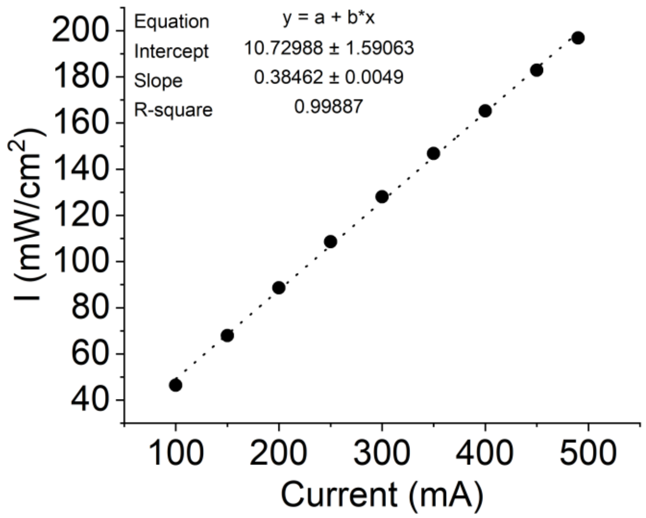

Fig. S17.

Intensity measurement for 455 nm LED light equipped with Kohler illumination setup.

**Monomer 1 composition**	**κ**
**9.75wt.%**	$\boldsymbol{-8.633 \times 10^{-7}T^4 + 1.930 \times 10^{-4}T^3 - 0.014 \times T^2 + 0.401 \times T - 4.622}$
**24.5wt.%**	$\boldsymbol{-1.792 \times 10^{-5}T^3 + 3.540 \times 10^{-3}T^2 - 0.159 \times T + 1.858}$
**97.5wt.%**	$\boldsymbol{2.056 \times 10^{-5}T^3 - 2.780 \times 10^{-3}T^2 + 0.138 \times T - 3.046}$

Table S1.

The curve fit formulas extracted from the experimental bending curvature data.

Movie S1.
Self-sustained oscillations of autonomous snapper.

Movie S2.
Influence of heat transfer area on self-sustained snapping behavior.

Movie S3.
Influence of surface temperature on self-sustained snapping behavior.

Movie S4.
Self-sustained oscillations of snappers with varying crosslink density.

Movie S5.
Demonstration of snapper's physical intelligence to detect color.
